# Highly Porous 3D Gold Enhances Sensitivity of Amperometric Biosensors Based on Oxidases and CuCe Nanoparticles

**DOI:** 10.3390/bios12070472

**Published:** 2022-06-29

**Authors:** Nataliya Stasyuk, Olha Demkiv, Galina Gayda, Andriy Zakalskiy, Halyna Klepach, Nina Bisko, Mykhailo Gonchar, Marina Nisnevitch

**Affiliations:** 1 Institute of Cell Biology National Academy of Sciences of Ukraine, 79005 Lviv, Ukraine; stasukne@nas.gov.ua (N.S.); demkivo@nas.gov.ua (O.D.); zakalskyae@nas.gov.ua (A.Z.); gonchar@cellbiol.lviv.ua (M.G.); 2 Department of Biology and Chemistry, Drohobych Ivan Franko State Pedagogical University, 82100 Drohobych, Ukraine; pavlishko@yahoo.com; 3 Institute of Animal Biology of the National Academy of Agrarian Sciences of Ukraine, 79034 Lviv, Ukraine; 4 M. G. Kholodny Botany Institute, National Academy of Sciences of Ukraine, 01601 Kyiv, Ukraine; bis-ko_nina@ukr.net; 5 Department of Chemical Engineering, Ariel University, Kyriat-ha-Mada, Ariel 4070000, Israel

**Keywords:** electroactive nanoparticles, peroxidase-like nanozyme, oxidases, micro/nanoporous gold, amperometric biosensors

## Abstract

Metallic nanoparticles potentially have wide practical applications in various fields of science and industry. In biosensorics, they usually act as catalysts or nanozymes (NZs) and as mediators of electron transfer. We describe here the development of amperometric biosensors (ABSs) based on purified oxidases, synthesized nanoparticles of CuCe (nCuCe), and micro/nanoporous gold (pAu), which were electro-deposited on a graphite electrode (GE). As an effective peroxidase (PO)-like NZ, nCuCe was used here as a hydrogen-peroxide-sensing platform in ABSs that were based on glucose oxidase, alcohol oxidase, methylamine oxidase, and L-arginine oxidase. At the same time, nCuCe is an electroactive mediator and has been used in laccase-based ABSs. As a result, the ABSs we constructed and characterized were based on glucose, methanol, methyl amine, L-arginine, and catechol, respectively. The developed nCuCe-based ABSs exhibited improved analytical characteristics in comparison with the corresponding PO-based ABSs. Additionally, the presence of pAu, with its extremely advanced chemo-sensing surface layer, was shown to significantly increase the sensitivities of all constructed ABSs. As an example, the bioelectrodes containing laccase/GE, laccase/nCuCe/GE, and laccase/nCuCe/pAu/GE exhibited sensitivities to catechol at 2300, 5055, and 9280 A·M^−1^·m^−2^, respectively. We demonstrate here that pAu is an effective carrier of electroactive nanomaterials coupled with oxidases, which may be promising in biosensors.

## 1. Introduction

Metallic nanoparticles have wide potential practical applications in various fields of science and industry. In biosensorics, they usually act as carriers, mediators in electron transfer, and/or catalysts (artificial enzymes or nanozymes) [1,2,3,4,5]. 

Nanozymes (NZs) are the newest class of functional nanomaterials [3,4,5,6,7]; they have enzyme-like activities with different reaction specificities. NZs possess increased stability and greater availability due to their simpler preparation technologies. Most reported NZs are mainly mimetics of oxidoreductases, including peroxidase (PO) [7,8,9,10,11].

PO catalyzes the oxidation of diverse organic compounds using H_2_O_2_ as the electron acceptor [8]. Many natural enzymes (oxidases) produce H_2_O_2_ as a byproduct of their enzymatic reactions, so the detection of a target substrate can be performed by measuring H_2_O_2_ generation. Over the last few years, a number of reports have described the application of various mimetics of PO for H_2_O_2_ detection using different sensors [8,9,10,11,12,13,14,15]. The main peculiarities of PO-like NZs as catalysts are that they have high stability, sensitivity, and selectivity to H_2_O_2_ in extra-wide linear ranges. PO-like NZs coupled with natural oxidases are widely used in electrochemical biosensors [7,8,9,16].

Oxidase-based amperometric biosensors (ABSs) are the simplest and most commonly employed type of biosensors. An enzyme-based ABS offers a means for quantitative analytical information; it can function via the measurement of signals in the form of current, which changes according to varying concentrations of the target analyte at a fixed potential. The main analytical characteristics of ABSs are the sensitivity, specificity, selectivity, detection limit, signal-to-noise ratio, linear dynamic ranges, and response time [1,2,17,18]. To effectively convert the biological response resulting from the interaction between analytes and enzymes into an electric current signal, the enzyme must be in direct spatial contact with the transducer. The selection of the appropriate methods to immobilize the enzyme on the electrode surface plays a significant role in the design of ABSs [1,2].

A number of approaches have been proposed for improving the analytical characteristics of ABSs, especially sensitivity. One of them is to increase the effective working surface of an electrode in order to obtain the maximal electroactive sites for the immobilization of biocatalysts, including enzymes and NZs [6,12,13,19,20,21,22,23]. In particular, 2D and 3D materials with extra-large areas are the promising candidates for this aim [1,17,18,19,20,21]. A lot of synthetic methods for obtaining 3D materials have been described [10,24,25,26,27,28,29,30].

Micro/nanoporous gold (pAu) is one of the best-studied 3D materials, and it has attracted increasing interest over the last twenty years [23,31,32,33,34]. pAu has a high surface-area-to-volume ratio, excellent conductivity, chemical inertness, physical and chemical stability, biocompatibility, electrochemical activity, easy tunability, and controllable pores as well as reduced stiffness and plasmonic properties. pAu possesses a higher roughness factor (the ratio between the real surface area and the geometrical area of the electrode) due to its porous structure. As a result, a pAu-modified electrode significantly increases the number of adsorption sites for enzymes and other biomolecules, thus improving electron transport in comparison with corresponding electrodes with nonporous surfaces. Thus, due to its intriguing properties, pAu is a very promising material for application in (bio)sensing, energy storage, diagnostic medicine, and drug delivery [14,19,21,22,23,24,35,36,37,38,39,40,41,42]. 

A variety of approaches to synthesize pAu have been reported in addition to methods of sputtering and self-assembling [17,27,41]. The pore size in pAu can be modulated from 5 up to 50 nm, depending on the type of synthesis protocol used [24,30].

Chemical and electrochemical dealloying were shown to lay a historical foundation for other methods [25,27,31,32]. This approach is appropriate for the fabrication of both monoporous (i.e., nanoporous or microporous) and hierarchically porous (i.e., possessing both microporosity and nanoporosity) metal structures with novel properties [23,24,43]. Hierarchical pores are highly desirable; the presence of larger-size pores enables the fast transport of the reactants, while the nanopores are responsible for providing a large surface area, thereby increasing the rate of electrochemical reactions. 

High-surface-area pAu (such as films, membranes, or powders) could also be designed by the electrodeposition technique. This method of pAu preparation on a solid substrate has been extensively researched, and it has become the most popular method in recent years [17,23,24]. The advantages of electrodeposition strategies include: one-step fabrication of thin films directly on a substrate; relatively easy control of particle morphology, size, and density; uniform deposition and good stability; and the formation of hierarchically porous metal structures with novel properties.

The currently known electrodeposition techniques able to form thin pAu films are: conventional electro-co-deposition in ionic liquids; lithographically patterned electrodeposition; electrochemical overpotential deposition; bicontinuous microemulsion; the pulse potentiostatic method; dynamic hydrogen bubble templates; and deep eutectic solvent-based deposition. 

In our earlier works, different types of PO-like NZs were synthesized and characterized [9,16]. The most electroactive nanoparticles of CuCe (nCuCe), having excellent sensitivity and a wide linear range for H_2_O_2_ detection, were used as effective artificial PO for the construction of L-arginine-sensitive ABS, which was based on L-arginine oxidase (ArgO) [16]. 

The aim of the current work was to demonstrate the crucial impact of pAu as an effective carrier of nanomaterials and enzymes on the analytical parameters of an ABS, especially on its sensitivity. The tasks of our study were to fabricate and characterize ABSs using various oxidases as biorecognition elements, the nCuCe as an electroactive functional nanomaterial, and the electrodeposited pAu with a highly advanced surface area as an effective carrier of oxidases and nCuCe.

## 2. Materials and Methods

### 2.1. Reagents

Cerium(III) chloride, copper(II) sulfate, L-arginine (Arg), methylamine, ethanol, methanol, *o*-dianisidine (*o*-DZ), hydrogen peroxide (30%), hydrogen tetrachloroaurumate(III) H[AuCl_4_], D-glucose, sodium sulfide, ammonia chloride, 2,2′-Azino-bis(3-ethylbenzthiazoline-6-sulfonic acid (ABTS), Nafion (5% solution in 90% low-chain aliphatic alcohols), horseradish peroxidase (PO, EC 1.11.1.7) from *Armoracia rusticana* (500 U∙g^−1^), and all other reagents and solvents used in this work were purchased from Sigma-Aldrich (Steinheim, Germany); glucose oxidase (GO, EC 1.1.3.4) from *Aspergillus niger* (168 U·mg^−1^) was purchased from Sigma-Aldrich (St. Louis, MO, USA). All reagents were of analytical grade and were used without further purification. All solutions were prepared using ultra-pure water obtained with the Milli-Q^®^ IQ 7000 water purification system (Merck KGaA, Darmstadt, Germany).

### 2.2. Enzymes, Isolation, and Purification

Purified enzymes—alcohol oxidase (AO, EC1.1.3.13), L-arginine oxidase (ArgO, EC 1.4.3.25), methylamine oxidase (AMO, EC 1.4.3.21), and laccase (EC 1.10.3.2)—were isolated by the authors from the corresponding sources and were used for the fabrication of amperometric biosensors (ABSs). Yeast AO was isolated from a cell-free extract of the selected overproducing strain *Ogataea polymorpha* C-105 *(gcr1 catX)* using a two-step ammonium sulfate fractionation (at 30% and 70% saturation), followed by ion exchange chromatography on a Toyopearl DEAE-650M [44]. Purified AO with the specific activity of ~20 U·mg^−1^ of protein was kept as a suspension in 70% sulfate ammonium (SA) and 50 mM phosphate buffer (PB) at pH 7.5 at 4 °C.

Mushroom ArgO was isolated from an extract of the fruiting body of the wild forest mushroom *Amanita phalloides* and partially purified up to ~7.9 U⋅g^−1^ of protein using a two-step SA fractionation (at 30% and 70% saturation), followed by chromatographic purification on a Toyopearl DEAE-650M [16]. Partially purified ArgO was kept as a suspension in 70% SA in 50 mM PB at pH 7.5.

The activities of AO, ArgO, or GO were determined by the rate of hydrogen peroxide formation in reactions with correspondent substrates (methanol [44], Arg [16], or glucose [10]) in the presence of PO and *o*-DZ in optimal conditions, which were chosen experimentally. After the incubation of reactants for a fixed time, the reactions were stopped by adding HCl. One unit of activity was defined as the amount of the enzyme required to oxidize 1 µmole of a substrate (ε_525_ = 13.35 mM^−1^·cm^−1^) per minute at 30 °C. The optical densities of these colored products were determined at 525 nm using a Shimadzu UV1650 PC spectrophotometer (Kyoto, Japan). 

Yeast AMO was isolated from the recombinant yeast strain *Saccharomyces cerevisiae* C13ABYS86 [45]. The (His)_6_-tagged AMO was purified from the cell-free extract by metal-affinity chromatography on Ni-NTA-agarose. The activity of AMO was determined by the rate of hydrogen peroxide formation in reaction with MA, as monitored by the peroxidative oxidation of ABTS in the presence of PO [45]. One unit of activity was defined as the amount of the enzyme required to oxidize 1 µmole of ABTS as a substrate (ε_420_ = 36 mM^−1^·cm^−1^) per minute at 30 °C.

Fungal laccase was purified from a cultural liquid of the fungus *Trametes zonatus* by a two-step SA fractionation (up to 70% of saturation), followed by chromatography on a Toyopearl DEAE-650M [46]. Fractions with the laccase activity were pooled, concentrated by a Millipore filter (10 kDa) up to a specific activity of enzyme ≥10 U·mg^−1^, followed by precipitation with 80% SA. The activity of laccase was determined by the rate of the increase in absorbance monitored spectrophotometrically at 420 nm. As a substrate, 0.5 mM ABTS in a 50 mM pH 4.5 sodium acetate (NaOAc) buffer solution was used. One unit of laccase activity was defined as the amount of the enzyme required to oxidize 1 µmole of ABTS per minute at 24 °C.

### 2.3. Synthesis of CuCe Nanoparticles and Estimation of Their Pseudo-Peroxidase Activity

Nanoparticles of CuCe (nCuCe) were synthesized and collected by centrifugation, as described previously [9]. The precipitates were rinsed twice with water and were stored as a water–colloid solution at +4 °C until use.

Pseudo-peroxidase (PO-like) activity of the nCuCe was measured using the colorimetric method with o-DZ as a chromogenic substrate in the presence of H_2_O_2_ [9]. One unit (U) of PO-like activity was defined as the amount of nCuCe consuming 1 µmol of H_2_O_2_ per 1 min at 30 °C under standard assay conditions. Here, we used a colloid solution of nCuCe with a PO-like activity of 1 U/mL.

### 2.4. Apparatus

A piece of Pt wire and an Ag/AgCI/3M KCI electrode were used as the counter and reference electrodes, and 3.05 mm graphite rods (type RW001, Ringsdorff Werke, Bonn, Germany) were used as working electrodes. The graphite electrode (GE) was prepared as described in detail previously [10]. Amperometric measurements were carried out with a CHI 1200A potentiostat (IJ Cambria Scientific, Burry Port, UK) in batch mode under continuous stirring in a standard 40 mL cell at a room temperature.

A REMMA-102-02 SEM microanalyzer (Lviv, Ukraine) was used for the morphological analyses of the synthesized porous gold.

### 2.5. Electrodeposition of Porous Gold onto Graphite Electrode

A layer of micro/nanoporous gold (pAu) was synthesized on the surface of a GE in two stages. In the first stage, pAu was electrodeposited from a solution containing 10 mM HAuCl_4_ in 2.5 M ammonia chloride using cyclic voltammetry in the range of 0 to +800 mV with a scan rate of 50 mV·min^−1^ for 25 cycles. In the second stage, the obtained modified electrode (pAu/GE) was re-immersed in a solution of 10 mM HAuCl_4_ in 2.5 M ammonia chloride using the potentiostatic mode at −1000 mV for 120 s. The obtained pAu/GE was rinsed with water and equilibrated before usage in the appropriate buffer.

### 2.6. Immobilization of nCuCe and Peroxidase on Electrodes

The synthesized nCuCe was immobilized on the surfaces of a GE and a pAu/GE, using the physical adsorption method. For this purpose, aliquots of nCuCe solution (10 μL) were dropped onto the surfaces of the GE and pAu/GE. 

For the development of the PO/GE, an aliquot of PO solution (10 μL) with an activity of 1 U/mL was dropped onto the surface of a GE. 

The modified electrodes were rinsed with 50 mM pH 7.5 PB and kept in this buffer with 0.1 mM EDTA at 4 °C until use.

### 2.7. Immobilization of Oxidases onto the Modified Electrodes

To fabricate the oxidase-based amperometric biosensors (ABSs), GO, AMO, AO, ArgO, or laccase were immobilized onto the modified GE.

First, 5–10 μL of enzyme solution was dropped onto the dried surfaces of the PO/GE, nCuCe/GE, or nCuCe/pAu/GE. The dried composites were covered with a Nafion membrane. To prepare a 1% Nafion solution, the stock 5% solution was diluted with the appropriate buffer: with 50 mM pH 4.5 NaOAc for the construction of laccase-based ABS and with 50 mM pH 7.5 PB in other cases.

It is worth mentioning that in the case of AO-based ABS, the biosensing film on the electrode was fixed with a dialysis membrane but not with Nafion.

The coated bioelectrodes were rinsed with water and stored in the corresponding buffers until use.

### 2.8. Measurements and Calculations

Amperometric measurements were carried out using a CHI 1200A potentiostat (IJ Cambria Scientific, Burry Port, UK) connected to a personal computer, which was used in a batch mode under continuous stirring in an electrochemical cell with a 20 mL volume at 25 °C.

All experiments were carried out in triplicate trials. The analytical characteristics of the proposed electrodes were statistically processed using OriginPro 8.5 software. Error bars represent the standard error derived from three independent measurements. The calculation of the apparent Michaelis–Menten constants (*K_M_^app^*) was performed automatically by this program according to the Lineweaver–Burk equation.

## 3. Results

### 3.1. Development of Oxidase-Based Biosensors Using nCuCe and Porous Gold

**Figure 1:**
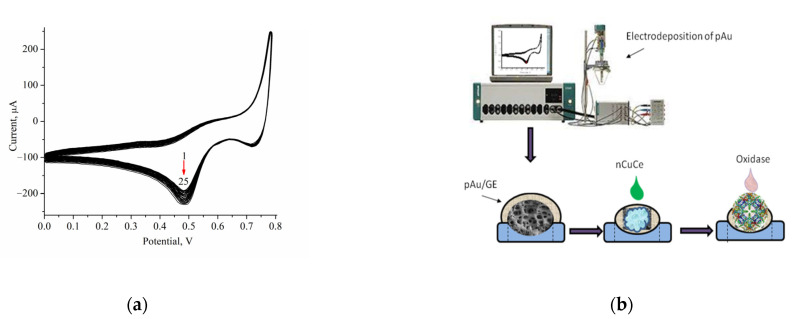
(**a**) CV profiles during 25 cycles of film electrodeposition with a scan rate 50 mV s^−1^ vs. Ag/AgCl; (**b**) Scheme of the working electrode preparation.

Micro/nanoporous gold (pAu) was reported to have a large area and a high surface-area-to-volume ratio; thus, it may be used to enhance the sensitivity of ABSs as carriers of enzymes [19,23,41]. We modified the surface of a graphite electrode (GE) with pAu. The electrodeposition of pAu onto the GE was carried out as described in Section 2.5. Figure 1a demonstrates the profiles of cyclic voltammograms (CVs) during the pAu film’s formation. We describe here the development of ABSs using oxidases coupled with nCuCe, which were immobilized on the surface of a pAu-modified GE. The principal scheme of bioelectrode construction is presented in Figure 1b.

A characteristic feature of all oxidases is the ability to catalyze oxidation reactions; namely, the transfer of electrons from electron donor to electron acceptor. As a result of these reactions, hydrogen peroxide (H_2_O_2_) is formed. For the effective functioning of oxidase-based ABSs, it is necessary to decompose H_2_O_2_ using natural or artificial peroxidase (PO). 

nCuCe plays a dual role in ABSs, being an effective artificial PO (PO-like NZ) [9,16] and, at the same time, an electroactive mediator of electron transfer.

**Figure A1:**
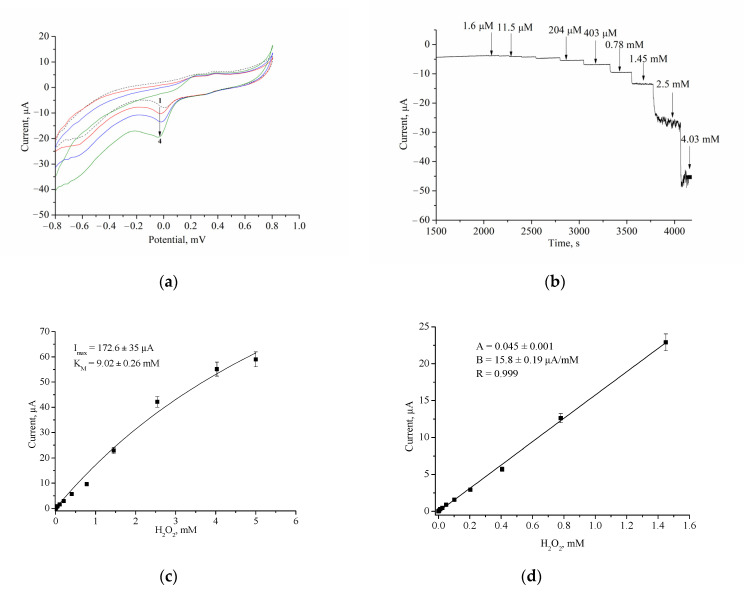
Amperometric characteristics of nCuCe/GE as a chemosensor of H_2_O_2_. (**a**) CV profiles as current responses upon addition of analyte up to concentrations (mM): 0 (1, black); 2.5 (2, red); 5.0 (3, blue); 10 (4, green). (**b**,**c**) chronoamperometric response and dependence of amperometric signal on concentration of H_2_O_2_, respectively; (**d**) calibration graph for H_2_O_2_ determination. Conditions: Ag/AgCl (reference electrode) in 50 mM PB, pH 7.0; scan rate 50 mV·s^−1^ (**a**); working potential —50 mV (**b**–**d**).

To demonstrate PO-like activity of the nCuCe/GE, *c*yclic voltammetry (CV) was used. These experiments were reported in detail in our previous papers [9,16]. The CV profiles of nCuCe/GE as current responses upon the addition of H_2_O_2_ are demonstrated in Figure A1a. According to the CV profiles, nCuCe/GE is sensitive to H_2_O_2_. Chronoamperometric experiments were also carried out (Figure A1b), and the calibration was performed by a stepwise addition of H_2_O_2_. Following the chronoamperograms, calibration graphs for H_2_O_2_ determination in wide and linear ranges were plotted (Figure A1c,d). These results proved that nCuCe is an artificial PO and nCuCe/GE may be an amperometric chemosensor of H_2_O_2_. The main analytical characteristics of the nCuCe/GE, namely, *K_M_^app^* and *I_max_*, are presented in Figure A1c. Other operational parameters, namely, the linear range (up to 1.5 mM) and limit of detection (0.5 µM), were determined from the calibration graph (Figure A1d). The sensitivity to H_2_O_2_ (2164 A·M^−1^·m^−2^) was calculated as a ratio of B (the parameter of linear regression of the calibration graph) to the surface area of a working electrode.

**Figure A2:**
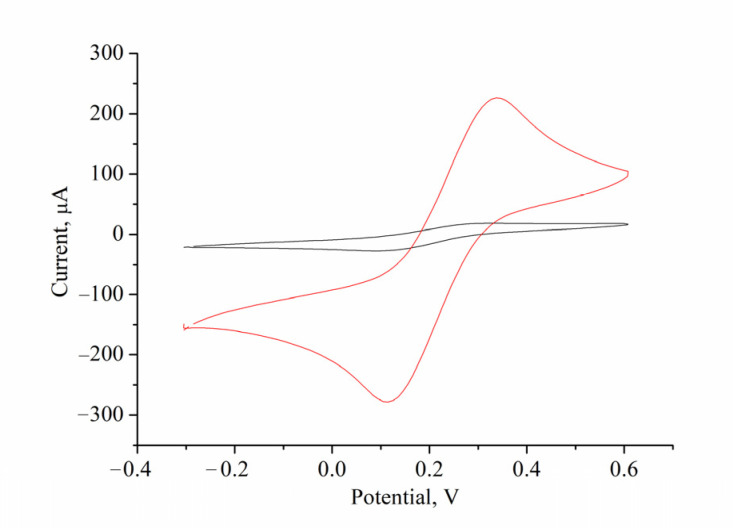
*C*yclic voltammetry (CV) study of the bulk GE (black) and the modified nCuCe/GE (red) in solution containing 10 mM K_3_Fe(CN)_6_, 100 mM KCl, and 50 mM NaOAc, pH 4.5.

The electroactivity of the nCuCe/GE was demonstrated using CV experiments in the presence of K_3_Fe(CN)_6_ (Figure A2). According to the results of the CV study, the tested nCuCe is electroactive since the peaks of oxidation and recovery of K_3_Fe(CN)_6_ on the nCuCe/GE were higher than those for the control unmodified GE.

**Figure 2:**
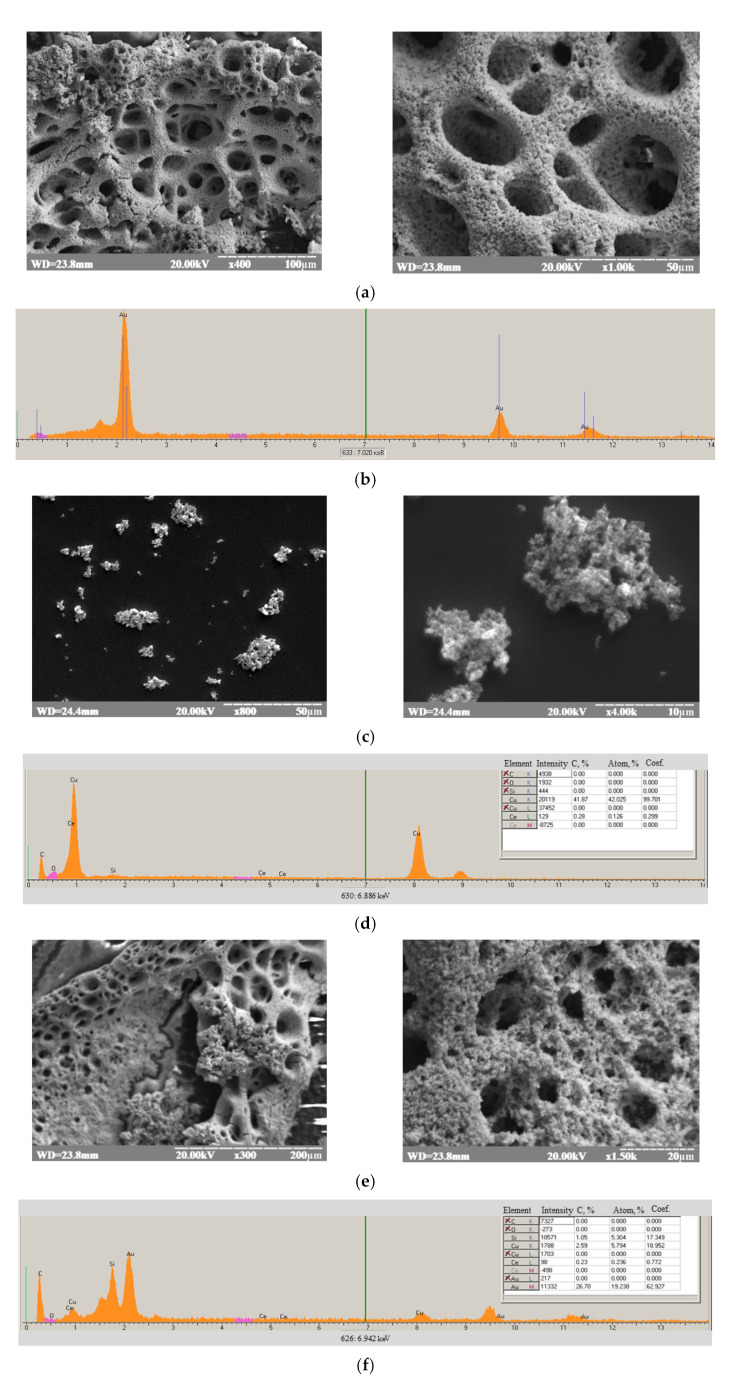
Characteristics of the pAu/GE (**a**,**b**), nCuCe/GE (**c**,**d**), and nCuCe/pAu/GE (**e**,**f**). SEM images with different magnitudes (**a,c,e**); X-ray spectral microanalysis (**b**,**d**,**f**).

Figure 2 demonstrates the results of the morphological characterization of pAu (Figure 2a), nCuCe (Figure 2c), and nCuCe/pAu (Figure 2e) using the SEM technique, which provides information on the size, distribution, and shape of the tested materials. 

The XRM images (Figure 2b,d,e) showed the presence of all components of the tested materials [9]. Au^0^ formation was proven by X-ray microanalysis, which showed the characteristic emission peaks at 2.1, 9.7, and 11.6 keV, corresponding to the AuK_α_, AuK_α_, and AuK_β_ transitions, respectively (Figure 2b). The characteristic peaks for Cu^0^ at 1.4 and 8.05 keV, which correspond to the CuK_α_ and CuK_β_ transitions, respectively, are shown in Figure 2d,f [47]. The peaks for Ce at 0.9 keV and 4.8 keV (Figure 2d,f) correspond to the CeK_α_ and CeK_β_ transitions [48]. 

### 3.2. Characterization of the Constructed Biosensors

**Figure 3:**
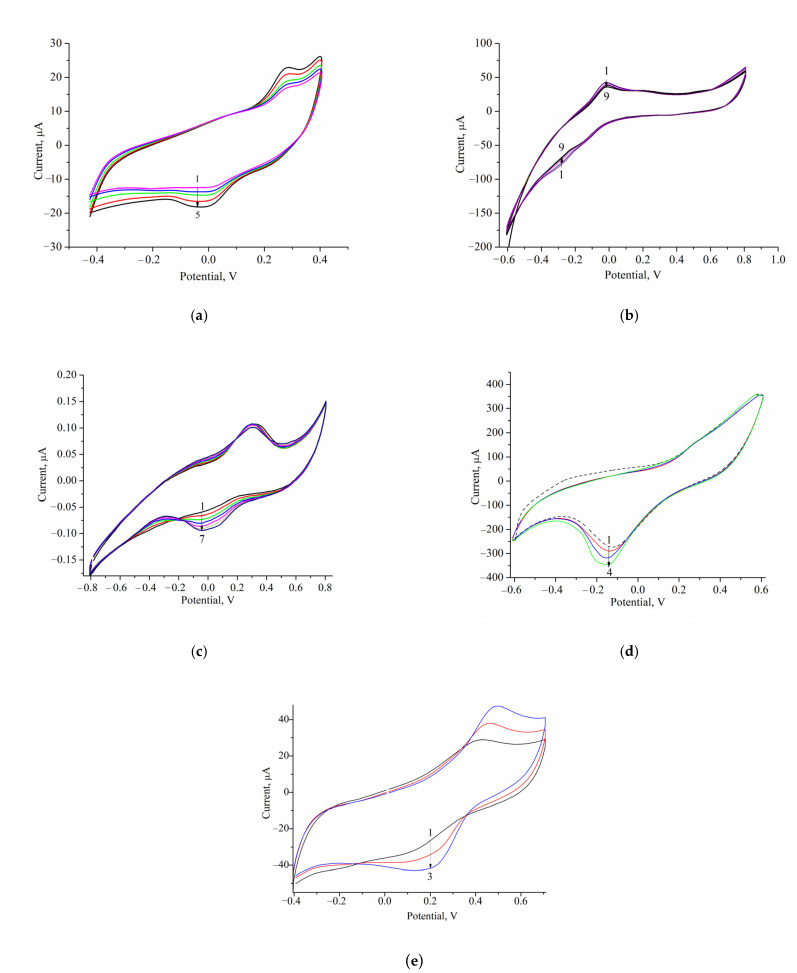
Profiles of CVs for the nCuCe/GEs, modified with GO (**a**), AMO (**b**), AO (**c**), ArgO (**d**), and laccase (**e**). Conditions: Ag/AgCl (reference electrode); 50 mM phosphate buffers (PB), pH 6.0 (**a**), pH7.5 (**b**–**d**), and NaOAc buffer, pH 4.5 (**e**); scan rate −50 mV·s^−1^. Substrates were added up to concentrations: 0–4 mM glucose, lines 1–5, respectively (**a**); 0–1 mM MA, lines 1–4, respectively (**b**); 0–2 mM methanol, lines 1–7, respectively (**c**); 0–13 mM L-Arg, lines 1–4, respectively (**d**); 0–1 mM catechol, lines 1–3, respectively (**e**).

**Figure 4:**
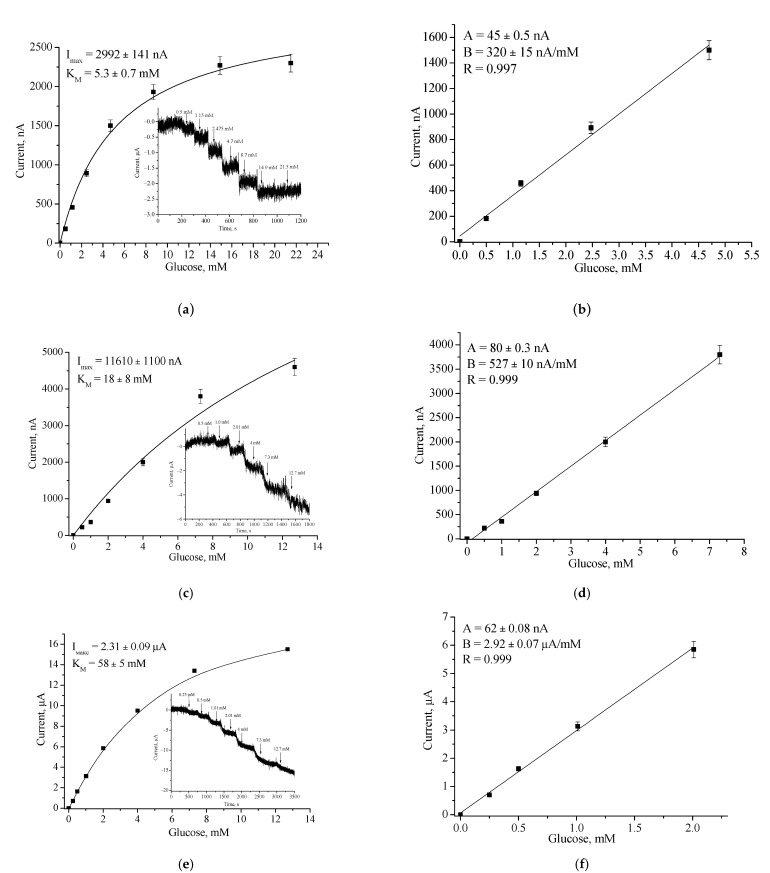
Amperometric characteristics of the GO/PO/GE (**a**,**b**), GO/nCuCe/GE (**c**,**d**), and GO/nCuCe/pAu/GE (**e**,**f**): (**a**,**c**,**e**) chronoamperograms (inserted) and dependence of amperometric signal on concentration of glucose; (**b**,**d**,**f**) calibration graphs for glucose determination. Conditions: working potential −50 mV vs. Ag/AgCl/3 M KCl in 50 mM PB, pH 6.0. The sensing layers contained 0.01 U of PO/PO-like activity and 0.01 U of GO.

**Figure 5:**
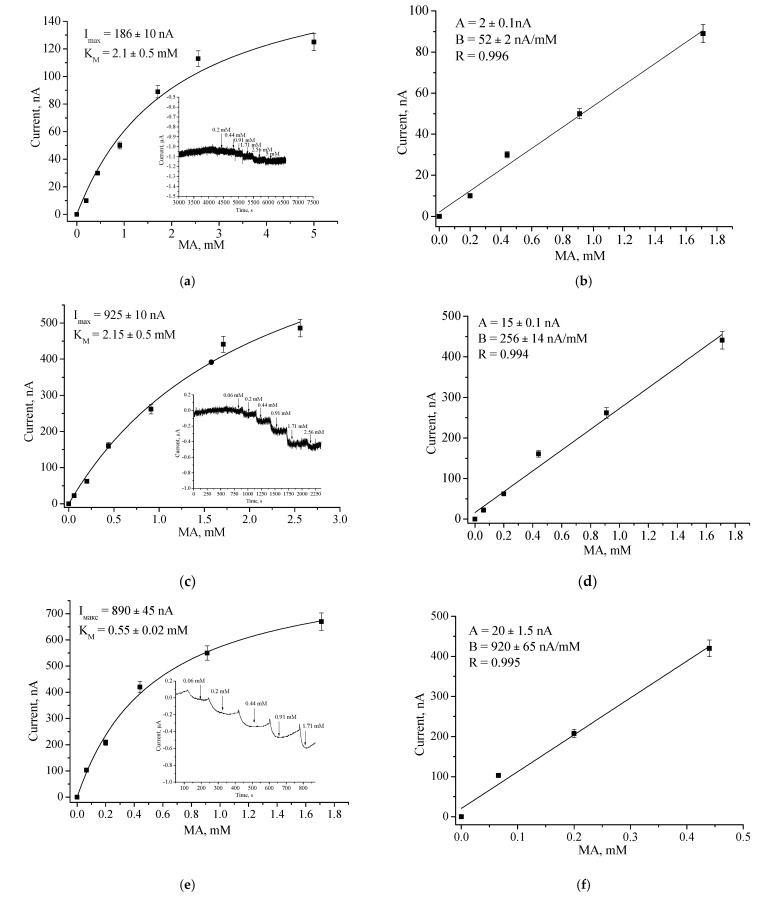
Amperometric characteristics of the AMO/PO/GE (**a**,**b**), the AMO/nCuCe/GE (**c**,**d**), and the AMO/nCuCe/npAu/GE (**e**,**f**): (**a**,**c**,**e**) chronoamperograms (inserted) and dependence of amperometric signal on concentration of MA; (**b**,**d**,**f**) calibration graphs for MA determination. Conditions: working potential −250 mV vs. Ag/AgCl/3 M KCl in 50 mM PB, pH 7.5.

**Figure 6:**
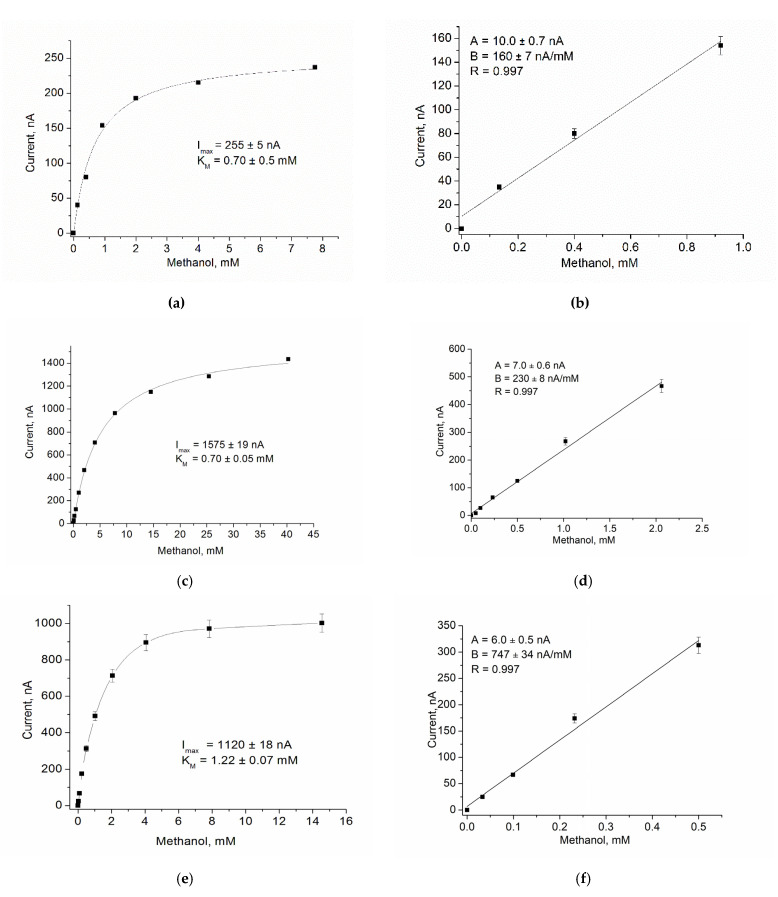
Amperometric characteristics of the AO/PO/GE (**a**,**b**), the AO/nCuCe/GE (**c**,**d**), and the AO/nCuCe/pAu/GE (**e**,**f**): (**a**,**c**,**e**) dependence of amperometric signal on concentration of methanol; (**b**,**d**,**f**) calibration graphs for methanol determination. Conditions: working potential −50 mV vs. Ag/AgCl/3 M KCl in 50 mM PB, pH 7.5.

**Figure 7:**
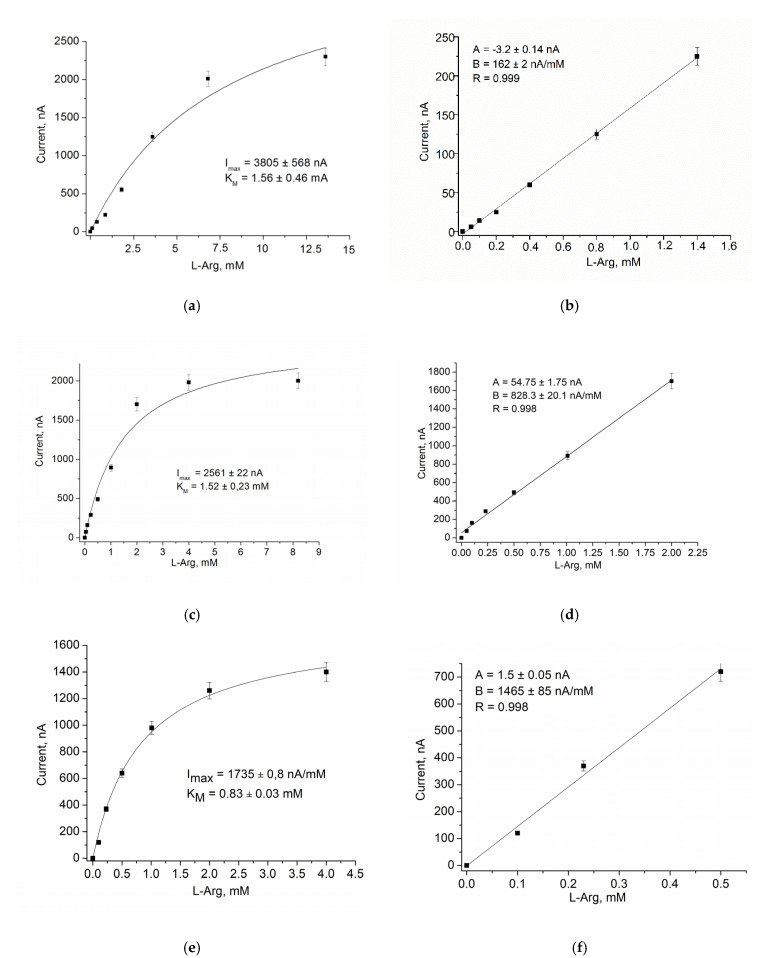
Amperometric characteristics of the ArgO/PO/GE (**a**,**b**), the ArgO/nCuCe/GE (**c**,**d**), and the ArgO/nCuCe/pAu/GE (**e**,**f**): (**a**,**c**,**e**) dependence of amperometric signal on concentration of L-Arg; (**b**,**d**,**f**) calibration graphs for L-Arg determination. Conditions: working potential −150 mV vs. Ag/AgCl/3 M KCl in 50 mM PB, pH 7.5.

**Figure 8:**
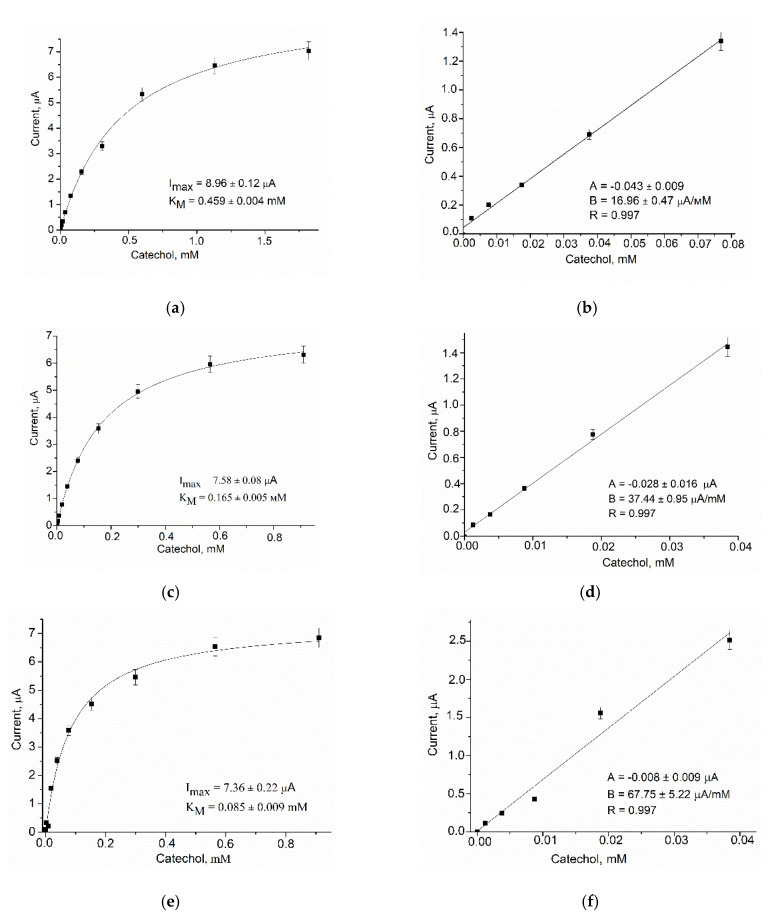
Amperometric characteristics of the laccase/GE (**a**,**b**), the laccase/nCuCe/GE (**c**,**d**), and the laccase/nCuCe/pAu/GE (**e**,**f**): (**a**,**c**,**e**) dependence of amperometric signal on concentration of catechol; (**b**,**d**,**f**) calibration graphs for catechol determination. Conditions: working potential –200 mV vs. Ag/AgCl/3 M KCl in 50 mM NaOAc, pH 4.5.

Using GO, AO, AMO, or ArgO as biorecognition elements, nCuCe as a PO-like NZ or as an electroactive mediator, and pAu as a carrier of enzymes/NZs, ABSs were constructed and characterized for glucose, primary alcohols, MA, Arg, and catechol, respectively (Figure 3, Figure 4, Figure 5, Figure 6, Figure 7 and Figure 8). 

**Figure A3:**
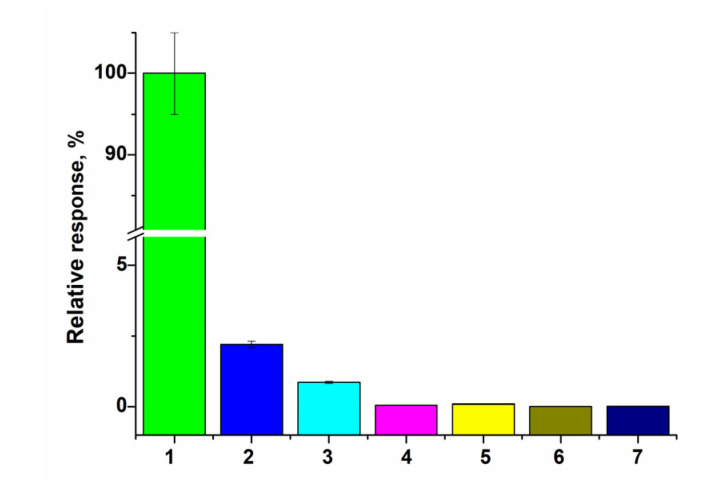
The selectivity test for the CuCe/GE: comparison of current responses on the added analytes up to 2 mM concentration: H_2_O_2_ (1), L-Arg (2), glucose (3), methylamine (4), methanol (5), ethanol (6), and catechol (7). Conditions: working potential −50 mV vs. Ag/AgCl (reference electrode), 50 mM acetate buffer, pH 4.5 at 23 °C.

It is worth mentioning that before modification with enzymes (see Section 2.7), the control electrodes were tested for their sensing of the target analytes (glucose, primary alcohols, methyl amine, Arg, and catechol). The results of the selectivity study for nCuCe/GE are shown in Figure A3. The selectivity of nCuCe/GE towards the tested analytes was estimated in relative units (%) as a ratio to the value of the highest response on addition of H_2_O_2_. We demonstrated the absence of any amperometric signals for the nCuCe/GE and other control electrodes (pAu/GE, nCuCe/pAu/GE, and PO/GE, data not shown) with each tested analyte addition under the chosen conditions. Therefore, nCuCe/GE may be an effective H_2_O_2_-sensitive platform for the construction of oxidase-based ABSs.

#### 3.2.1. Optimal Working Potential

The efficiency of electron transfer from oxidase to the surface of the electrode (GE) and the selection of the optimal potentials for each enzyme were evaluated by a cyclic voltammetric technique. The CV profile is the dependence of the current response on changing potentials under increasing concentrations of the appropriate substrate. Figure 3 demonstrates the CVs of the GEs that were modified with oxidase and nCuCe. 

#### 3.2.2. Analytical Properties

Chronoamperometric studies were carried out under the chosen optimal potentials for each oxidase-based electrode. Three types of modified electrodes were studied, namely, enzyme in combination with a natural PO (designated as oxidase/PO/GE), with CuCe as an artificial PO (oxidase/nCuCe/GE), and with CuCe on the surface of a pAu-modified electrode (oxidase/nCuCe/pAu/GE). Taking into account the chronoamperograms at optimal working potentials, calibration curves for the modified and control electrodes were plotted for analyte determination by the developed ABSs (Figure 4, Figure 5, Figure 6, Figure 7 and Figure 8). 

Figure 4 demonstrates the amperometric characteristics for the GO-based ABS for glucose determination. The optimal potential for the developed GO/nCuCe/GE is −50 mV (see Figure 3a); thus, the calibration of working electrodes was carried out under this potential using a solution of 50 mM glucose.

The same experiments were carried out for all other developed ABSs, which contained AMO, AO, ArgO (Figure 5, Figure 6 and Figure 7), and laccase (Figure 8). 

Table 1 summarizes the main bioanalytical characteristics of the developed ABSs, which were based on the usage of pAu, various oxidases, and nCuCe as well as the optimal conditions for their exploitation. It is worth mentioning that nCuCe plays a dual role in the described ABSs: for laccase it is a mediator of electron transfer, and for the other oxidases it is an artificial PO.

## 4. Discussion

In the present work, the development of ABSs based on different oxidases and nCuCe, which were co-immobilized on pAu, was described. nCuCe has a dual role as an active mimetic of PO and a mediator of electron transfer. It was used as an electro-active mediator for a laccase-based ABS and as a PO-like NZ in ABSs that were based on other oxidases, namely, GO, AO, AMO, and ArgO. The ABSs for determination of catechol, glucose, primary alcohols, methyl amine, and L-arginine were constructed and characterized. The developed mono-enzyme ABSs exhibited improved analytical characteristics in comparison with the correspondent bi-enzyme ABSs that contained natural PO. 

As shown in Table 1, nCuCe had a significant positive effect on sensor sensitivity in comparison to electrodes that were not modified with a nanomaterial. For example, for AMO/nCuCe/GE and ArgO/nCuCe/GE, the sensitivities were 5-fold higher (S_2_) than for the corresponding GEs with natural PO (S_1_). The ABS that contained laccase/nCuCe/GE was 2.2-fold more sensitive than the ABS with the laccase/GE composition. 

It was demonstrated that the impact of electrodeposited pAu in the chemo-sensing layer of a graphite electrode is also significant. The presence of pAu was shown to provide an additional contribution to improve the analytical parameters of the ABS, especially in terms of their sensitivities (see Table 1). For example, the sensitivity of the GO/nCuCe/pAu/GE is 9.1-fold higher than that of the GO/PO/GE and 5.5-fold higher in comparison to the GO/nCuCe/GE. The same tendency, but at various levels, was demonstrated for each investigated enzyme. Thus, the ratio of sensitivities (S_3_/S_2_) for the AO-based ABS had a value of 3.2; for the AMO-based ABS, this value was 3.6. The positive influence of pAu on the sensitivity of an ABS has a simple explanation: the highly advanced surface of the pAu, having hierarchical pores with different diameters, presents an enhanced working 3D surface area on the electrode. The increased surface of the modified GE leads to the enhanced adsorption of nanomaterials/enzymes and thus to improved efficiency of electron transfer in the ABS in comparison with unmodified GEs.

Thus, ABSs with 3D porous micro/nanomaterials are novel versions of these devices that demonstrate a number of advantages, including enhanced sensitivity and stability, in comparison with traditional ABSs. The 3D architecture of the ABSs led to the improved analytical characteristics of the electrodes because of the increased amounts of immobilized enzyme and the enhanced speed of electron transfer. For successful progress in the development of ABSs to the level of industrial fabrication of commercially viable products, further integration of sensing technology with biochemical approaches needs to be achieved. 

## Figures and Tables

**Table 1:** Analytical characteristics of the constructed bioelectrodes.

Bioelectrode				Potential, mV	Sensitivity (S)		Linear Range, µM	LOD, µM	*K_M_^app^*, mM
Enzyme	Mr, kDa	Sensor for H_2_O_2_	N		A·M^−1^·m^−2^	K_S_			
AO	640	PO	1	–50	22	S_2_/S_1_ = 1.5	130–900	39	0.70
		nCuCe	2		32	S_3_/S_2_ = 3.2	50–2100	15	0.70
		nCuCe/pAu	3		102	S_3_/S_1_ = 4.6	33–500	10	1.22
AMO	160	PO	1	–250	7	S_2_/S_1_ = 5	200–1700	61/130	2.1
		nCuCe	2		35	S_3_/S_2_ = 3.6	60–1700	18	2.2
		nCuCe/pAu	3		125	S_3_/S_1_ = 17.9	60–450	18	0.55
ArgO	500	PO	1	–150	22	S_2_/S_1_ = 5.1	75–1400	35	1.56
		nCuCe	2		113	S_3_/S_2_ = 1.8	50–2250	15	1.52
		nCeCu/pAu	3		200	S_3_/S_1_ = 9.1	100–500	33	0.83
GO	150–190	PO	1	–50	44	S_2_/S_1_ = 1.7	500–5000	150	5.23
		nCuCe	2		73	S_3_/S_2_ = 5.5	500–7300	150	18
		nCeCu/pAu	3		400	S_3_/S_1_ = 9.1	250–2000	76	5.81
Laccase	100	bulk	1	+200	2300	S_2_/S_1_ = 2.2	8–160	2	0.46
		* nCuCe	2		5055	S_3_/S_2_ = 1.8	3–40	2	0.17
		nCuCe/pAu	3		9280	S_3_/S_1_ = 4.0	2–40	1	0.09

* nCuCe is a mediator of electron transfer here.

## Data Availability

The data are contained within the present article.

## Appendix A

Appendix A presents amperometric characteristics of nCuCe/GE, namely, data of its PO-like properties (Figure A1), electroactivity (Figure A2), and selectivity (Figure A3).
